# Gun-Shot Wound of the Chest Treated by Hermetically Sealing

**Published:** 1864-10

**Authors:** P. F. Browne

**Affiliations:** Surgeon in Charge First Division Chimborazo Hospital.


ART. V.—*Gun-Shot Wound of the Chest Treated by Hermetically Sealing.* Reported by P. F. BROWNE, Surgeon in Charge 1st Division Chimborazo Hospital.

Private J. W. Branson, company "C.," 9th Virginia cavalry, was wounded July 29th, 1864, by a minie ball, which entered the right side of the chest, about three inches below the axilla, traversed the right lung, and was removed by counter-incision near the spinal column on a level with the orifice of entrance.

There was bleeding from the lung at the time the wound was received—he was brought directly from the field to the hospital, and in less than twenty-four hours after the wound was received He was a strong, athletic man, about twenty-seven years of age. It was decided, as the wound had been so recently received, and his condition every way favorable, to treat the case by hermetically sealing the wound. The orifice of entrance was carefully closed by means of thin layers of cotton, saturated with collodion; these were renewed as often as found necessary to prevent introduction of air in the chest—in a word, the wound was kept hermetically sealed. The case progressed favorably, attended by only slight circumscribed inflammation of the lung, some effusion in the pleural cavity and a little fever. These were so slight as to require but little treatment. The patient, at the time of his reception, and for several weeks afterwards, complained of great soreness of both sides of the chest—more of the left than the right; this soreness was increased by the slightest pressure. He attributed, and I think justly, this soreness to a severe fall from his horse at the time he was wounded. Under appropriate treatment and rest the soreness gradually subsided, and the patient when fairly convalescent and walking about the ward was furloughed, and left in a carriage for his home in Westmoreland county, Virginia, with every reason to expect a speedy and perfect recovery.

How far the favorable result in this case is attributable to the treatment pursued, I will not undertake to decide. I report the case to accumulate facts on which fair and safe deductions may hereafter be drawn of the success of treating gun-shot wounds of the chest by "hermetically sealing."

*Ligation of the External Iliac for Traumatic Aneurism of the Femoral Artery.*

Private B. B. Brown, company "C.," 9th Virginia cavalry, was wounded June 24th, 1864, by a small ball, apparently a buckshot or pistol ball, which entered the left groin just below Pouparts ligament, grazing the femoral artery on its outer side, and passing entirely through the ramus of the pubis near the ilio pectineal eminence. The patient was a strong, healthy man, about twenty-four years of age. When admitted into division No. 1, July 3d, he complained of no pain at the seat of injury of any consequence, except at night. The paroxysm came on regularly, shortly after sundown.

There was but little pain on pressure in the immediate neighborhood of the wound; no tenderness over the abdomen, and no appreciable swelling at the mouth of the wound, which presented a very healthy appearance. He had no fever; appetite very good, and bowels moved regularly every day. There was, however, considerable soreness and pain on pressure on the inner side of the thigh, just below the origin of the gracilis muscle, and for which there was no satisfactory means of accounting, as there was no swelling and no visible sign of inflammation at that spot.

All efforts to introduce a probe into the wound were unavailing—it would follow the track of the ball to the bone where it met with a resistance that could not be overcome.

He continued to do well till the morning of July 14th, when a pulsating tumor was discovered at the seat of injury, small, but having the distinct thrill and bruit characteristic of aneurism. The tumor increased rapidly in size, and it was determined to try the effect of digital compression—ten men were, therefore, detailed, and instructed how to keep up pressure just above the aneurism, with a key well padded, relieving each other every half hour. Finding this inefficient and not to be depended on, a compressor was constructed, consisting of an upright firmly attached to the side of the bed, with a horizontal limb projecting, to which a second piece was attached by an iron bolt, and pierced by two holes to accommodate two large wooden screws. These screws were furnished with oval blocks covered with kid tightly drawn. One screw pressed on the cardial side and one on the distal side of the tumor, and relieved each other at regular intervals.

By this arrangement the circulation could be perfectly restrained. Pressure was kept up for three days and nights, allowing a very small stream to pass through the wounded vessel. At the expiration of this time the skin began to slough to such an extent that the pressure had to be discontinued. The tumor had not diminished, though it had not increased in size. The thrill and bruit were as well marked as ever and the pulsation very plain. It then became a necessity to ligate the external iliac at once. The operation was performed July 18th, by Assistant-Surgeon J. C. Baylor.

A curvilinal incision four and a half inches in length, beginning one and a half inches above Poupart's ligament, and near the internal abdominal ring, and ending opposite to the superior anterior spinous process of the ilium.

Not an ounce of blood was lost during the operation, and no difficulties of any moment were met with at any step. One very small subcutaneous artery was divided by the first incision, and instantly secured by a ligature. The peritoneum was healthy in appearance, and was easily pushed aside to make room for the application of the ligature. The artery was tied about its middle. All pulsation in the tumor ceased as soon as the ligature was fastened. The wound was then brought together by interrupted suture, extended down through the muscles and fascia, so as to bring all parts of the wound in apposition. He was then carried back to bed, the leg placed in a flexed position, and forty drops tr. opii. administered.

Next morning, July 19th, he expressed himself as feeling very comfortable. Had passed a very good night. He ate his breakfast with much relish; pulse 120. There was some swelling and puffiness about the incision, and slight pain on pressure over the abdomen in its immediate neighborhood. July 20th.—Suffers no pain; puffiness and swelling around the incision much diminished; considerable soreness, but extending only a few inches from the wound; quite a free discharge of muddy pus; pulse 110. He was ordered an enema of ol ricini and soapsuds. July 21st.—Doing very well; pus healthy; discharge from the wound quite free; pulse 110; bowels moved slightly by the enema. July 23d.—Condition very favorable; sleeps well; appetite good; soreness very much diminished; swelling disappeared; pulse 100. July 25th.—Continues to do well; appetite very good; bowels regular; no soreness on pressure over the abdomen; wound granulating finely; pulse 95; July 28th.—Pulse 112; has suffered much for the last two days from involuntary contractions and starting of the wounded limb; appetite good; wound looks well, and healing rapidly.

From this time till August 8th the case progressed very favorably. An amount of irritability was perceptible in the pulse, however, that the healthy appearance of his wound did not justify, and could not be accounted for. The incision healed up very prettily. Ligature came away on the 15th day; very slight discharge of pus, not more than a teaspoonful in twenty-four hours. Quite an extensive slough had taken place in two spots where the screws had pressed; the lower one was a deep excavation, caused by the suppuration of several of the inguinal glands. This place had commenced to fill up, and presented a clean and healthy appearance.

On August 8th, while his wounds were being dressed, a sudden and very free discharge of urine, mingled with pus, burst from the excavation, and as such an accident was entirely unexpected, it created much surprise. On questioning the patient closely he mentioned, for the first time, that he had passed blood very freely from the urethra shortly after he was wounded, and that a catheter had been kept in the bladder the whole night by the surgeon on the field. Urine continued to ooze during the day very freely, and a catheter was introduced and fastened in the bladder. This diminished the flow of urine from the wound, but caused so much irritation that it had to be withdrawn.

After the removal of the catheter he passed urine through the urethra several times a day, while the flow from the wound gradually decreased till, at the end of a week, it passed almost exclusively through the natural channel.

A large bedsore had formed, in the meantime, over the sacrum, and from this also urine began to flow. He now lost flesh rapidly. His pulse became very weak and frequent. Stimulants were freely administered to him, but he sank, and died August 22d, 1864.

This case presents many points of interest:

1st. The failure of compression to relieve the aneurism through digital and instrumental means were successively employed.

2d. The entire success of the operation, and obliteration of the aneurismal tumor by ligation of the external iliac.

3d. There was no recurrent hemorrhage, though the patient lived thirty-five days after the operation.

4th. There was no diminution of warmth in the leg.

5th. The death of the patient from sloughing of the bladder and discharge of urine through the wound, caused evidently by the ball at the time the wound was received. No autopsy could be held, and the exact course of the ball of course remains unknown, as also the amount of injury inflicted by it on the internal parts.

